# The Anti-inflammatory Effect of the Tricyclic Antidepressant Clomipramine and Its High Penetration in the Brain Might Be Useful to Prevent the Psychiatric Consequences of SARS-CoV-2 Infection

**DOI:** 10.3389/fphar.2021.615695

**Published:** 2021-03-09

**Authors:** B. Nobile, M. Durand, E. Olié, S. Guillaume, J. P. Molès, E. Haffen, P. Courtet

**Affiliations:** ^1^ Department of Emergency Psychiatry and Acute Care, CHU Montpellier, Montpellier, France; ^2^ IGF, Univ. Montpellier, CNRS, INSERM, Montpellier, France; ^3^ Pathogenesis and Control of Chronic Infection, University of Montpellier, INSERM, EFS; CHU Montpellier, Montpellier, France; ^4^ FondaMental Foundation, Créteil, France; ^5^ Service de Psychiatrie de l’Adulte, CIC-1431 INSERM, CHU de Besançon, Laboratoire de Neurosciences, Université de Franche-Comté, Besancon, France

**Keywords:** SARS-CoV-2, COVID-19, antidepressants, clomipramine, inflammation, psychiatric pathology

## Abstract

At the time of writing (December 2020), coronavirus disease 2019 (COVID-19) has already caused more than one million deaths worldwide, and therefore, it is imperative to find effective treatments. The “cytokine storm” induced by Severe Acute Respiratory Syndrome-Coronavirus type 2 (SARS-CoV-2) is a good target to prevent disease worsening, as indicated by the results obtained with tocilizumab and dexamethasone. SARS-CoV-2 can also invade the brain and cause neuro-inflammation with dramatic neurological manifestations, such as viral encephalitis. This could lead to potentially incapacitating long-term consequences, such as the development of psychiatric disorders, as previously observed with SARS-CoV. Several pathways/mechanisms could explain the link between viral infection and development of psychiatric diseases, especially neuro-inflammation induced by SARS-CoV-2. Therefore, it is important to find molecules with anti-inflammatory properties that penetrate easily into the brain. For instance, some antidepressants have anti-inflammatory action and pass easily through the blood brain barrier. Among them, clomipramine has shown very strong anti-inflammatory properties *in vitro*, *in vivo* (animal models) and human studies, especially in the brain. The aim of this review is to discuss the potential application of clomipramine to prevent post-infectious mental complications. Repositioning and testing antidepressants for COVID-19 management could help to reduce peripheral and especially central inflammation and to prevent the acute and particularly the long-term consequences of SARS-CoV-2 infection.

## Introduction

The global pandemic of the new coronavirus disease 2019 (COVID-19) caused by Severe Acute Respiratory Syndrome-Coronavirus type 2 (SARS-CoV-2) started in Wuhan, China, in December 2019, and has spread rapidly worldwide, due to its high transmissibility (Munster et al., 2020; Zhang et al., 2020b). The majority of infected people have mild symptoms. However, about 20% of them develop a severe form with high mortality (Wu and McGoogan, 2020). In view of the time needed to develop specific antiviral drugs (several months if not years) and the urgent need of efficient therapeutics, drug repositioning seems to be the best way to prevent COVID-19 complications. Repositioning, defined as the new use of a drug in addition to its original indications, is now considered the fastest way to find efficient treatments (Nicol et al., 2020; Sanders et al., 2020; Serafin et al., 2020). It is also crucial to find efficient therapeutics to prevent COVID-19 long-term consequences. Indeed, during the one year of this pandemic, different long-term consequences of SARS-CoV-2 infection have been described, including psychiatric disorders (Taquet et al., 2020).

Although COVID-19 pathophysiology is not well known yet, the growing body of work already available sheds some light on the mechanisms involved in the infection and paves the way for the discovery of potential therapeutics. Currently, hundreds ongoing clinical trials are testing different drugs that might target various features of the disease physiopathology (Thorlund et al., 2020). Among these different aspects, excessive inflammation following SARS-CoV-2 infection is an important target. Indeed, the worsening of infected patients is mainly explained by the amplified immune response and cytokine release, also called “cytokine storm” (Felsenstein et al., 2020; Sanders et al., 2020). This phenomenon involves an important release of pro-inflammatory cytokines, such as interleukin 6 (IL-6) and tumor necrosis factor α (TNFα) (Li et al., 2013; Sanders et al., 2020). It has been hypothesized that the cytokine storm contributes to the respiratory deficiency observed in these patients by increasing the alveolar–capillary blood–gas exchange dysfunction that eventually leads to pulmonary fibrosis and organ failure (Xu X. et al., 2020; Xu Y.-H. et al., 2020). Targeting the cytokine storm with tocilizumab (a humanized anti-IL-6 monoclonal antibody) or dexamethasone has shown promising effects in reducing COVID-19 severity and mortality in severely ill patients (Sanders et al., 2020; WHO Rapid Evidence Appraisal for COVID-19 Therapies (REACT) Working Group et al., 2020; Xu X. et al., 2020). On the other hand, it is now recognized that SARS-CoV-2 infection leads also to neurological damage, either caused by the direct invasion of the Central Nervous System (CNS) and/or indirectly through the neuro-inflammation induced by the infection (Marshall, 2020). Approximately 40% of patients with COVID-19 have neurological symptoms (e.g., confusion, agitation) (Helms et al., 2020; Mao et al., 2020; Richardson et al., 2020). More worrying, this virus can cause encephalitis with poor prognosis (Carod Artal, 2020; Moriguchi et al., 2020). It has been hypothesized that the cerebrovascular and neuronal damage caused by the disease could contribute to its severity, notably to the respiratory deficiency (Li et al., 2020). In addition, virus-induced neurological damage could lead to long-term consequences in COVID-19 survivors (Troyer et al., 2020). Some patients have cognitive impairment (sometimes severe) after COVID-19 (Zhou et al., 2020), and some patients infected by SARS-CoV-2 (sometimes up to about 50%) develop psychiatric disorders after recovery (Mazza et al., 2020; Taquet et al., 2020; Varatharaj et al., 2020; Zhang et al., 2020a). Although the mechanisms underlying the development of psychiatric problems following SARS-CoV-2 infection are not known, recent studies suggest that COVID-19 psychiatric consequences are related to inflammation, as indicated for instance by the positive correlation between presence of depressive symptomatology and cytokine levels (Guo et al., 2020; Mazza et al., 2020; Yuan et al., 2020). This is in agreement with previous research showing that inflammation due to some viral infections could lead to psychiatric disorders (Wright et al., 1995; Buckley, 2019; Pape et al., 2019). Peripheral inflammation induced by a viral infection can cause indirectly neuro-inflammation, and some viruses can directly induce neuro-inflammation. As the mechanisms whereby SARS-CoV-2 induces brain disorders are not known, it is important to find molecules with anti-inflammatory properties (peripheral and central) that pass easily through the blood brain barrier (BBB), diffuse largely in the CNS at therapeutic concentrations, and might prevent the psychiatric consequences of SARS-CoV-2 infection.

The finding that deregulation of the inflammatory response (e.g., increase of pro-inflammatory cytokines) is involved in depression pathophysiology (Su, 2012; Kohler et al., 2016), among other mechanisms, led to study the potential anti-inflammatory properties of antidepressant drugs. Antidepressant molecules, especially some selective serotonin reuptake inhibitors and tricyclic antidepressants (TCAs), have anti-inflammatory properties and diffuse easily in the CNS. Among them, clomipramine, a TCA that acts mainly by inhibiting serotonin and noradrenaline reuptake (Balant-Gorgia et al., 1991), displays a certain and reproducible anti-inflammatory effect (Baumeister et al., 2016), particularly in the CNS (Faissner et al., 2017). Due to its action on inflammation (peripheral and central), its facility to penetrate and accumulate in the CNS and its antidepressant and anti-anxiety properties, clomipramine could be a potential prophylactic treatment to prevent COVID-19 psychiatric sequelae, for instance, in moderately to severely ill patients.

The aim of this mini-review is to build on our previous work suggesting that clomipramine could be of potential use in preventing COVID-19 neurological complications (Nobile et al., 2020) and to discuss the wider therapeutic implications of clomipramine in this infectious disease. First, we will describe possible mechanisms of the cytokine storm induced by SARS-CoV-2. Next, we will review literature data on clomipramine effects on peripheral inflammation (in serum). Then, we will present data on SARS-CoV-2 effects on the CNS and how clomipramine might prevent them through its anti-inflammatory action in the CNS and its global mechanism of action.

### Possible Mechanisms of the “Cytokine Storm” in Blood Induced by SARS-CoV-2

RNA viruses, such as SARS-CoV-2, that infect host cells are targeted by the innate antiviral response, mainly driven by interferon type-I (IFN-I) molecules, such as IFN-α and IFN-β. Viral RNA is considered a Pathogen Associated Molecular Pattern (PAMP) that can initiate the production and release of IFN-I and pro-inflammatory cytokines (IL-1, IL-6, TNFα), through the nuclear factor κB (NF-κB) and interferon regulatory factor 3 (IRF3) pathways. Upon recognition of viral RNA as an intruder by RIG-I-like receptors (RLRs), different mechanisms are triggered in infected cells, leading to activation of the NF-κB and IRF3/7 pathways, and resulting in the recruitment of immune T cells and IFN-I production, respectively (Frieman et al., 2008; Chen et al., 2017). IL-1, IL-6 and TNFα further promote pro-inflammatory molecule production mainly through the NF-κB pathway. All these mechanisms result in the activation and recruitment of immune cells and clearance of viral infection through inflammation. However, in some cases, this phenomenon, the aim of which is to protect tissues from viral infection, can get out of control with deleterious consequences for cells and tissues. For reasons still not completely understood, in some patients with COVID-19, the innate immune response is not properly regulated, resulting in the massive production of pro-inflammatory cytokines, with harmful effects (Loo and Gale, 2011; Choudhary et al., 2021). This condition has been named “cytokine storm” or cytokine release syndrome (Jin et al., 2017; Swanson et al., 2019). The cytokine storm clinical manifestations are the direct consequences of the disproportionate and systemic inflammatory response: hyper-permeability and excessive coagulation, high fever, asthenia, coagulopathy, thrombosis, Acute Respiratory Distress Syndrome (ARDS), multi-organ failure, and death. In patients with COVID-19, the cytokine storm increases alveolar damage and promotes pulmonary edema, leading to pulmonary injuries and ARDS (Tay et al., 2020; Choudhary et al., 2021).

Moreover, some viruses can escape the antiviral innate system through inhibition of the IFN-I production pathway. SARS-CoV and MERS-CoV, two coronaviruses that display high similarities with SARS-CoV-2 and belong to the same family (Betacoronaviruses) (Rabaan et al., 2020), have this capacity. SARS-CoV can alter RLRs and inhibit IFN-I production and immune cell recruitment (Astuti and Ysrafil, 2020). Nevertheless, at a later infection stage, the massive death of host cells due to viral infection leads to the release of pattern recognition receptors (PRRs), composed of viral particles and cellular debris. This results in the late and sudden innate immune response stimulation, through massive release of pro-inflammatory cytokines and recruitment of T and B-lymphocytes. This might contribute also to the deleterious self-powered loop, leading to the cytokine release syndrome and its harmful consequences (Felsenstein et al., 2020).

In patients with COVID-19, high concentrations of pro-inflammatory cytokines and other inflammatory markers have been associated with severe prognosis, acute respiratory distress syndrome, and multi-organ failure (Ye et al., 2020). Indeed, high levels of IL-2R (the receptor of the anti-inflammatory cytokine IL-2 that plays a key role in regulating the immune response; high concentrations have been associated with excessive immune response (Yang and Lundqvist, 2020)) and IL-6 have been significantly correlated with the infection severity. Some studies also found that patients with COVID-19 in intensive care units have high concentrations of inflammatory markers, such as TNFα, underlying the inflammation response deregulation (Ye et al., 2020). In addition, acute respiratory distress syndrome is mainly caused by massive lung infiltration by inflammatory cells (monocytes, macrophages and lymphocytes), resulting in alveolar damage and pneumocyte hyperplasia (Felsenstein et al., 2020). Interestingly, lung inflammation is higher following viral clearance, with a peak after about 14 days of disease, supporting the hypothesis of an excessive activation of the immune system (Clay et al., 2012; Felsenstein et al., 2020).

It is important to note that the cytokine storm following SARS-CoV-2 infection is more likely to occur in patient with chronic inflammation (Nidadavolu and Walston, 2020). In agreement, patients with chronic diseases (e.g., cardiovascular diseases, diabetes, obesity, chronic lung diseases) are more susceptible to SARS-CoV-2 severe infection (Yang et al., 2020). The higher risk of severe infection in older people might also be related to immunosenescence and immune system impairment (e.g., decreased activity of anti-inflammatory cytokines) (Vellas et al., 2020). Environmental factors (e.g., pollution, smoking) also contribute to enhance risk of negative outcomes (Alqahtani et al., 2020). Genetic factors also could be implicated, as previously shown for the TLR1 polymorphism and Gram-positive bacterial infections and for a single polymorphisms in the *IFN-l3* gene and hepatitis C virus infection (Thomas et al., 2009). Altogether, these data indicate that susceptibility to excessive immune activation is multifactorial and that inflammation induced by SARS-CoV-2 should be a key target to prevent complications.

### Peripheral Anti-Inflammatory Properties of Clomipramine

As noted, it is important to target the COVID-19-related peripheral inflammation and cytokine storm to prevent COVID-19 complications in general, and also in the brain. Indeed, the peripheral and the central immune systems interact, and even a mild dysfunction of the peripheral immune system could lead to an alteration of the central one (Buckley, 2019). Many studies reported that the TCA clomipramine has anti-inflammatory properties. The link between inflammatory deregulation and depression led researchers to evaluate the anti-inflammatory properties of antidepressants (e.g., amitriptyline, fluoxetine) (Kubera et al., 2013; Alcocer-Gómez et al., 2014), with contradictory results related to the study type (e.g., fluoxetine decreased IL-6 levels in one study and increased it in another one) and used concentrations (e.g., desipramine at low doses stimulates and at high doses inhibits IL-10 and IFN-γ) (Baumeister et al., 2016). Clomipramine was one of the few antidepressants (with amitriptyline) showing anti-inflammatory properties in all studies. Its anti-inflammatory properties could be partly mediated by effects on signaling cascades activated during viral infection, such as the NF-κB pathway (Hwang et al., 2008), and also on the mitochondrial respiratory chain (Rooprai et al., 2003). Hence, we decided to focus this mini-review on this antidepressant.

In the late 1990s, *in vitro* studies on clomipramine demonstrated its anti-inflammatory properties. A study on monocytes and T lymphocytes found that clomipramine significantly reduces IL-1β, IL-6 and TNFα production by monocytes and also IFNγ and IL-2 production by T lymphocytes (Xia et al., 1996). Moreover, a study on diluted whole blood, to preserve cell-to-cell interactions, showed that clomipramine decreases IFNγ production and increases that of IL-10, an anti-inflammatory cytokine that inhibits IL-6 and TNFα synthesis (de Waal Malefyt et al., 1991). These effects were observed at therapeutic plasma concentrations (Maes et al., 1999).

Several studies tried to elucidate the mechanisms of clomipramine anti-inflammatory effect. Diamond et al. (Diamond et al., 2006), using cells isolated from human blood samples, demonstrated that clomipramine suppresses the production of cytokines (e.g., IFNγ) by T cells (Th1), increases IL-10 production, and decreases IL-1β production by monocytes. They found that the reduced production of pro-inflammatory cytokines was explained by clomipramine inhibition of T-cell proliferation. An *in vivo* model to evaluate the anti-inflammatory effect of antidepressants showed that clomipramine reduces the inflammatory exudate induced by carrageenan or dextran injection in the rat paw (two methods to induce different inflammatory response types) (Abdel-Salam et al., 2003; Gurgel et al., 2013; Kostadinov et al., 2014). This effect could results from clomipramine interference with the activity of inflammatory mediators, such as histamine, serotonin and bradykinin (Abdel-Salam et al., 2003), but also through clomipramine-mediated reduction of neutrophil migration and mast cell stabilization (Gurgel et al., 2013). The authors suggested that this effect could be due to clomipramine tricyclic chemical structure because amitriptyline (another TCA) has the same effect, and because non-tricyclic antidepressants do not act directly on neutrophil migration (Sacerdote et al., 1997, 1994). In a recent study, Kostadinov et al. (Kostadinov et al., 2014) demonstrated that after carrageenan injection, clomipramine (single dose or repeated doses) inhibits edema formation in the rat paw and TNFα and IL-6 production, but increases Transforming Growth Factor 1 β (TGF1 β) and IL-10 production. They suggested that this effect was partially linked to clomipramine activity on serotonin levels. Indeed, it was previously reported that high extracellular levels of serotonin decrease TNFα and IL-6 production (Kubera et al., 2005), and that clomipramine increases serotonin extracellular levels. Clomipramine also modulates human glucocorticoid receptor function in whole human blood samples, and this might partly explain its anti-inflammatory effects (Carvalho et al., 2010). All the mechanisms underlying clomipramine anti-inflammatory properties have not been elucidated yet, and clomipramine may exert its anti-inflammatory actions through some [e.g., effects on mitochondria in glioma (Higgins and Pilkington, 2010)] or all of these pathways. Nevertheless, the anti-inflammatory efficacy of clomipramine at therapeutic concentrations has been proven and replicated in many *in vitro* and *in vivo* studies.

Concerning studies in humans, some antidepressant drugs significantly reduce the plasma levels of IFNγ, IL-1 β, IL-6 and TNFα in depressed patients (Frommberger et al., 1997; Mikova et al., 2001; Strawbridge et al., 2015; Szałach et al., 2019). Two meta-analyses on this topic showed that 1) these effects are not observed with all antidepressants; 2) results vary according to the study (e.g., anti-inflammatory effect in a study but not in another study); and 3) not all antidepressants decrease the levels of all pro-inflammatory cytokines (e.g., some antidepressants only decrease IL-1 β level) (Hannestad et al., 2011; Hiles et al., 2012). Nevertheless, previous *in vitro* studies showed that some antidepressants, especially clomipramine and fluoxetine, more consistently decrease pro-inflammatory cytokines (e.g., IFNγ, IL-1 β, IL-6) (Baumeister et al., 2016). Therefore, due to its capacity to reduce peripheral inflammation, clomipramine might be a good candidate for reducing inflammation in patients with COVID-19, and might contribute to prevent cerebral complications. Moreover, an *in vivo* study in rats found that the highest concentrations of clomipramine (outside the brain) were in lungs and liver (Aitchison et al., 2010); therefore, it could be useful for reducing lung inflammation.

### Effects of SARS-CoV-2 on the CNS and Potential Protective Effects of Clomipramine

SARS-CoV-2 infection can also have a direct impact on the CNS. For instance, a Chinese study found that about 40% of patients hospitalized for SARS-CoV-2 infection have neurological symptoms (e.g., dysgeusia, anosmia, headache … ) (Mao et al., 2020). More worrying, SARS-CoV-2 can also cause encephalitis, leading to brain inflammation and lesions (Wu et al., 2020), and infectious toxic encephalopathy caused by respiratory distress and hypoxia. Furthermore, this viral infection can contribute to ischemic events and cerebrovascular accidents (Helms et al., 2020; Wu et al., 2020). Besides these short-term effects, COVID-19 might have long-term consequences, especially psychiatric disorders.

Several mechanisms can explain the short-term COVID-19 CNS symptoms (Marshall, 2020; Postolache et al., 2020). First, SARS-CoV-2 might directly infect brain cells where it replicates and impairs their functions. In addition to lung tropism, SARS-CoV-2 also shows an affinity for the CNS, as suggested by its presence in the cerebrospinal fluid of some infected patients (Khodamoradi et al., 2020; Moriguchi et al., 2020). Furthermore, brain tissue edema with partial neuronal degeneration was detected in patients who died due to infection by another coronavirus (i.e., SARS-CoV) (Xu et al., 2005). It has been hypothesized that coronaviruses enter the brain following the olfactory nerves, which could also explain the loss of smell (Butowt and von Bartheld, 2020; Troyer et al., 2020; Wu et al., 2020). Then, reduction of oxygen supply due to lung injury might lead to CNS hypoxia, cerebral edema, and even coma. Some viruses can infect macrophages, microglia or astrocytes and therefore, deregulate the brain inflammatory system (Soung and Klein, 2018). The uncontrolled inflammatory response can affect immune cells in the brain, such as glial cells, and induce or enhance brain inflammation (Wu et al., 2020). Furthermore, SARS-CoV-2 affinity for the Angiotensin Converting Enzyme 2 (ACE 2) receptor could abnormally increase blood pressure, thus increasing the risk of cerebral hemorrhage (Wang et al., 2020). The virus may also damage the BBB and enter the CNS by attacking the vascular system (Baig et al., 2020). Finally, some coronaviruses can spread via a synapse-connected route to the medullary cardiorespiratory center from the mechanoreceptors and chemoreceptors in the lung and lower respiratory airways (Wu et al., 2020). This suggests that the respiratory deficiency observed in COVID-19 could also have a central origin (Li et al., 2020). These are possible mechanisms to explain the “direct” and acute effects of CNS invasion by SARS-CoV-2.

Other mechanisms could also be implicated in such long-term consequences. Here, we will focus on the possible psychiatric sequelae of COVID-19 (Bouças et al., 2020; Pantelis et al., 2020). Previous studies already highlighted a possible link between some viral infections and psychiatric diseases, for instance hepatitis C virus and risk of depression (Adinolfi et al., 2017). Other coronaviruses have already been associated with the appearance of psychiatric disorders, such as psychosis, major depression and bipolar disorders (Cheng et al., 2004; Okusaga et al., 2011; Severance et al., 2011). A very recent study found that being infected by SARS-CoV is associated with a 2.8-fold higher risk of psychiatric disorders and suicide during the 12 years of follow-up (Tzeng et al., 2020). This could be an indirect effect of the high inflammation induced by the infection (Brietzke et al., 2020). As said previously, inflammation (peripheral and in the brain) has been linked to many psychiatric disorders (Brundin et al., 2015; Solmi et al., 2015; Courtet et al., 2016; Bollen et al., 2017) thus its effect on the CNS has been studied. In recent years, much research has focused on neuro-immunity to better understand the pathogenesis of psychiatric disorders. The interactions between neurons, glial cells and immune system contribute to cognitive functions and social behaviors (Pape et al., 2019). Consequently, even a mild dysregulation of one of these systems (e.g., the immune system) might facilitate the emergence of psychiatric disorders (Loonen and Ivanova, 2016; Buckley, 2019). For instance, it has been shown that an increase in pro-inflammatory cytokine levels reduces serotonin bioavailability (Baumeister et al., 2016), inhibits dopamine synthesis (Felger and Lotrich, 2013; Baumeister et al., 2014), increases glutamate release from astrocytes (resulting in higher concentration of extracellular glutamate that could lead to excitotoxic effects) (Haroon et al., 2017), alters the hypothalamic–pituitary–adrenal axis and the kynurenine pathway (Malek et al., 2015; Erhardt et al., 2017) that can also affect CNS function, and modulates the expression of factors involved in neuroplasticity (e.g., brain-derived neurotrophic factor) (Lima Giacobbo et al., 2019). Dysregulation of these systems has been associated with depression, bipolar depression, and suicidal behavior (Dell’Osso et al., 2016; Erhardt et al., 2017; Olié and Courtet, 2017).

Alternatively, it has also been hypothesized that SARS-CoV-2 affinity for ACE 2 receptors could lead to a decrease in serotonin and dopamine levels (Nataf, 2020). Indeed, ACE 2 is co-expressed with dopa decarboxylase, an enzyme of the dopamine and serotonin synthetic pathways (Nataf, 2020). By downregulating ACE 2 expression, SARS-CoV-2 also downregulates this enzyme and contributes to decreasing dopamine and serotonin levels, as previously reported for SARS-CoV (Kuba et al., 2005; Klempin et al., 2018). This could increase the risk of psychiatric disorders in vulnerable patients. All SARS-CoV-2 effects (direct and indirect) on the CNS are serious and should encourage the search of therapeutic molecules that pass through the BBB, with high bioavailability in the brain, anti-inflammatory properties, and that might prevent neurotransmitter (i.e., serotonin and dopamine) depletion, such as psychotropic drugs.

In addition to its certain peripheral anti-inflammatory properties, some studies demonstrated that clomipramine has anti-inflammatory effect in the CNS. Zhu et al. (Zhu et al., 1998) showed that in Lewis rats, clomipramine (at therapeutic concentrations) significantly reduces the symptoms of experimental autoimmune neuritis, a CD4-positive T-cell-mediated autoimmune disease characterized by inflammation and demyelination and a validated animal model for the study of Guillain-Barre syndrome, a human autoimmune disease. The authors also found that clomipramine reduces IFNγ production. Another study showed that in microglial cells co-cultured with neurons and incubated with lipopolysaccharides to induce acute inflammation, clomipramine at therapeutic concentrations decreases the production of TNFα and nitric oxide, and the mRNA expression of inducible nitric oxide synthase, IL-1 β and TNFα as well as the activation of the NF-κB and p38 MAPK pathways (Hwang et al., 2008). It also reduced cell death. These findings are very interesting because microglia and astrocytes are the main mediators of neuroinflammation. Microglia (about 10% of all glial cells) are the primary immune cells in the CNS, and are implicated in inflammation-mediated neurotoxicity (Liu and Hong, 2003) through the production of pro-inflammatory cytokines and neurotoxic mediators (e.g., TNFα, IL-1 β, IL-6). Astrocytes are the most abundant glial cell type in the CNS and upon inflammatory stimulation, they proliferate and produce various mediators, such as nitric oxide and TNFα (Clark et al., 2019). Moreover, a combined *in vitro* and *in vivo* study showed that clomipramine inhibits the nucleotide-binding oligomerization domain leucine-rich repeat-containing family pyrin domain-containing 3 (NLRP3) inflammasome (Gong et al., 2019), leading to a significant decrease of TNFα, IL-1 β, IL-6 levels and IL-1 β and IL-6 gene expression. Interestingly, the nicotinic receptor pathway plays a role in modulating the inflammatory response (notably via the α7 nicotinic acetylcholine receptors-α7nACh receptor) (Hoover, 2017; Andersson, 2020), and a study in *Xenopus* laevis showed that clomipramine can regulate nicotinic receptors (López-Valdés et al., 2002). Although clomipramine effect on nicotinic receptors in humans is not known, this finding might be interesting because the nicotinic system plays a role in cognitive functions and psychiatric disorders (Djemil et al., 2020; Venkatesan et al., 2020) by modulating the release of some neurotransmitters and neuroplasticity. Similarly, it has recently been hypothesized that ketamine anti-depressive effect is partially mediated by its action on the α7nACh receptor (Zhao et al., 2020). Moreover, SARS-CoV-2 might interact with nicotinic receptors (Changeux et al., 2020; Gonzalez-Rubio et al., 2020). Although this interaction and its role in the inflammatory process have not been elucidated yet, we could hypothesize that clomipramine contributes to inflammation modulation also by inhibiting the interaction between SARS-CoV-2 and nicotinic receptors. Additional studies are needed to determine the mechanisms underlying the interaction between SARS-CoV-2, nicotinic receptors, and clomipramine. Recently, Faissner et al. suggested that clomipramine could be a candidate anti-inflammatory molecule for the treatment of progressive multiple sclerosis (a brain inflammatory disease) (Faissner et al., 2017). This group analyzed the potential anti-inflammatory activity of 249 drugs against iron toxicity in human neurons in culture, and found that clomipramine was one of the molecules with the best effect. When they tested its anti-inflammatory property in *vivo* models of experimental autoimmune encephalomyelitis, they found that clomipramine has antioxidant effects and decreases T-cell proliferation and lymphocyte activation, at standard clinical doses.

In conclusion, clomipramine could be a candidate drug for preventing brain damage and particularly psychiatric disorders caused by SARS-CoV-2. In addition to its anti-inflammatory properties, clomipramine is a serotonin and noradrenaline reuptake inhibitor and therefore, it may partially treat a potential reduction in serotonergic neurotransmission associated with SARS-CoV-2 infection.

## Summary and Perspectives

The available knowledge on COVID-19 indicates that it is important to treat the acute phase of the disease, but also to keep in mind that this infection might cause long-term sequelae in survivors. Although anti-inflammatory molecules, such as dexamethasone, significantly decrease inflammation in patients with COVID-19 (WHO Rapid Evidence Appraisal for COVID-19 Therapies (REACT) Working Group et al., 2020), it is not known whether they can prevent neurological sequelae and especially the development of psychiatric disorders. Furthermore, as these anti-inflammatory drugs are mainly used in severely ill patients (i.e., patients hospitalized in intensive care units), no information is available on their efficacy in patients with mild-moderate symptoms who represent a large population at higher risk of psychiatric disorders upon SARS-CoV (Tzeng et al., 2020) and SARS-CoV-2 (Taquet et al., 2020) infection. Therefore, new specific anti-viral drugs and repositioned drugs are necessary. In view of the data reviewed here, clomipramine seems to be a good candidate because this molecule has reproducible anti-inflammatory properties and could prevent the brain damage caused by the direct effect of the viral infection and indirectly through the excessive inflammatory response. Moreover, clomipramine could limit serotonin depletion that may be caused by SARS-CoV-2.

Other antidepressants have anti-inflammatory properties, but we focused on clomipramine for various reasons. First, clomipramine has consistently shown anti-inflammatory properties in all studies. Second, clomipramine significantly decreases brain inflammation and has been proposed as a potential treatment for progressive multiple sclerosis, a severe autoimmune disease. Finally, in studies that screened the ability of large panels of molecules to inhibit virus replication, clomipramine significantly inhibited replication of Ebola virus (a RNA virus), SARS-CoV, and MERS-CoV (Dyall et al., 2014; Kouznetsova et al., 2014; Johansen et al., 2015; Dyall et al., 2017). The underlying mechanisms were not investigated. On the basis of the chemical structure of clomipramine, which is a cationic amphiphilic drug, we could hypothesize that it accumulates in lysosomes where it increases their pH (Vater et al., 2017), thus inhibiting the viral protease activation. More studies are needed to assess and confirm its action on viral replication and to identify the mechanisms involved.

Several studies demonstrated the anti-inflammatory properties of clomipramine at therapeutic plasma concentrations. Moreover, clomipramine enters easily the brain (probability to cross the BBB of 0.979 (Faissner et al., 2017)) and accumulates in this tissue (12.5-fold higher concentration than in plasma or serum levels) (Weigmann et al., 2000). Clomipramine is on the list of the essential medicines of the World Health Organization (World Health Organization, 2019), demonstrating its safety of use and its importance. Clomipramine can have side effects (like all drugs), including weight increase, sexual dysfunction, sedation, hypotension, and anticholinergic effects (dry mouth, sweating, obstipation, blurred vision, and micturition), but is globally well tolerated (Faissner et al., 2017). However, its anticholinergic effects require further investigations, especially in the context of SARS-CoV-2 infection. Clomipramine has already been assessed in healthy volunteers (without psychiatric pathology), and was well tolerated without mood changes (e.g., mania) at the doses used for patients with mood disorders (Cardoso de Almeida et al., 2010; Cerqueira et al., 2014).

In conclusion, the potential beneficial effects of clomipramine for preventing the deleterious consequences of SARS-CoV-2 infection need to be assessed. Other antidepressants that are better tolerated also could have anti-inflammatory action (i.e., selective serotonin reuptake inhibitors). Their anti-inflammatory properties should be thoroughly evaluated *in vitro* and *in vivo* before considering them as candidate repositioned drugs for treating SARS-CoV-2 infection. It may also be possible to envisage collaborative studies between psychiatrists (who routinely prescribe these molecules), virologists, immunologists and intensive care specialists to assess the potential effects of repurposed psychotropic medications.

## Data Availability

The original contributions presented in the study are included in the article/Supplementary material, further inquiries can be directed to the corresponding author.
